# COVID-19 Vaccination Compliance and Associated Factors among Medical Students during an Early Phase of Vaccination Rollout—A Survey from Israel

**DOI:** 10.3390/vaccines10010027

**Published:** 2021-12-27

**Authors:** Maayan Katz, Maya Azrad, Daniel Glikman, Avi Peretz

**Affiliations:** 1 The Baruch Padeh Medical Center, Clinical Microbiology Laboratory, Poriya, Tiberias 1528001, Israel; maayan645@gmail.com (M.K.); mazrad@poria.health.gov.il (M.A.); 2 Azrieli Faculty of Medicine, Bar-Ilan University, Safed 1311502, Israel; DGlikman@poria.health.gov.il; 3 The Baruch Padeh Medical Center, Infectious Diseases Unit, Poriya, Tiberias 1528001, Israel

**Keywords:** medical students, COVID-19, vaccine hesitancy, Israel, survey

## Abstract

COVID-19 is “a once-in-a-century” pandemic, bringing with it unparalleled health, social, and economic ramifications. As part of the world’s efforts to restrain the pandemic, vaccine development has been expedited. This population-representative survey in Israel aimed to investigate whether the knowledge, attitudes, and vaccination status of medical students affect their intention to recommend COVID-19 vaccination (as well as reasons for refusal and acceptance of the vaccine). The questionnaire was anonymous, via Google Forms app in December 2021. One-hundred and four medical students completed the survey. Overwhelmingly, (91.3%) COVID-19 vaccination status and intention to receive the vaccine were positively associated with intention to recommend. Twenty-five percent of the students replied that they lacked knowledge regarding the vaccine. A statistically significant association was found between experiencing quarantine and the intention to be vaccinated (*p* = 0.034). There was a significant positive relationship between the number of symptoms from previous vaccines and the fear of COVID-19 (rs = 0.272, *p* < 0.01). Prior vaccination did not have an effect on COVID-19 vaccine hesitancy. This first study evaluating COVID-19 vaccine hesitancy among Israeli medical students highlighted the need for medical programs to emphasize the benefits of COVID-19 vaccination in the protection of healthcare workers and patient safety. Education, awareness campaigns, and regulation of vaccine trials could further decrease COVID-19 vaccine hesitancy and increase vaccine rates among medical students.

## 1. Introduction

COVID-19 is described as a-once-in-a-century pandemic, bringing with it unparalleled health, social, and economic ramifications. Its rapid spread and high mortality rates have resulted in 250 million cases and 5 million fatalities worldwide as of November 2021 [1]. Only one year after COVID-19 was declared as a global emergency, vaccine development was completed. Mass vaccination against COVID-19 has emerged as a key preventive strategy [2].

Vaccinations against COVID-19 with the BNT162b2 mRNA vaccine were rolled out in Israel starting December 2020, and soon became part of an unprecedented campaign launched by the Israeli Ministry of Health and the health maintenance organizations (HMOs). The vaccination campaign began in risk factor groups (including persons over the age of 60) and medical staff only and was rapidly expanded to include the whole population over the age of 16 by the beginning of February 2021 [3]. To date (October 2021) Israel ranks 16 in national COVID-19 vaccination rate worldwide with 65% of the population fully vaccinated [1].

The use of a new vaccine technology, alongside a relatively rapid development process, have raised public concerns about the safety of the vaccine. A growing number of people worldwide believe that these vaccines may be harmful. The anti-COVID-19 vaccination movement has been driven by the lack of knowledge or incorrect data, as well as by concerns with regards to vaccine safety. These knowledge gaps were not limited solely to individuals without medical education but were also identified among medical students worldwide [4,5].

Medical students, soon to become physicians, are integral to raising community awareness, improving vaccination education, and developing campaigns to increase vaccination uptake among the general population and other healthcare workers. University students can play a leading role in public health service. Previous evidence showed that university students can comprise a core group helpful in addressing vaccine hesitancy through promoting positive attitude towards vaccination [4].

Physicians have a critical role in influencing vaccination attitudes and their recommendations are one of the strongest correlates of vaccine acceptability among patients [5]. In order to do so, healthcare providers must be confident about the safety and effectiveness of COVID-19 vaccines [2,5]. The current study investigated whether the knowledge, attitudes, and vaccination status of medical students in northern Israel influenced their intention to recommend COVID-19 vaccination (as well as reasons for refusal or acceptance of the vaccine).

## 2. Materials and Methods

### 2.1. Study Design

This study was conducted during December 2020 among medical students at the Azrieli Faculty of Medicine of Bar-Ilan University, Safed, Israel. The study was approved by the Institutional Review Board of the Faculty. The timing of the survey was in close proximity to the decision of the Israeli Ministry of Health to vaccinate medical students as part of the first wave of COVID-19 vaccinations. Data collection was entirely anonymous, with no individual identifying information.

The online questionnaire was developed using prior studies on vaccine hesitancy among medical students [6,7,8]. It was designed to collect information regarding basic demographic details, awareness and sources of information regarding COVID-19 vaccines, attitudes regarding the vaccine and prior, non-COVID-19 vaccination experience. The questionnaire was given online using Google forms, and its link was shared by social media of medical students. No reward was offered to students who completed the survey. Upon completion of the survey, data were collected, and analysis was conducted.

### 2.2. Statistical Analysis

Based on χ^2^ test, with the dependent variable “intention to be vaccinated” and the independent variables “pro vaccine attitude”, a moderate effect size 0.3, *α* = 0.05, and 0.85 power, the minimum sample size needed for the study was 88. The calculation was conducted using G*Power software (ChristianAlbrechts-Universität, Olshausenstr, Germany) [9].

The formula for calculating the sample size is described below:

(1) $$N=\frac{2{\left\{Z_{1-\frac{\alpha }{2}}\sqrt{2PQ}+Z_{1-\beta }\sqrt{P_{1}Q_{1}+P_{2}Q_{2}})\right\}}^{2}}{{\left(P1-P2\right)}^{2}}$$

The calculation was based on the proportions 0.5 (intention to be vaccinated among anti vaccine group) and 0.8 (intention to be vaccinated among pro vaccine group):

(2) $$N=\frac{2{\left\{1.96\sqrt{2*0.65*0.35}+1.04\sqrt{0.5*0.5+0.8*0.2})\right\}}^{2}}{{\left(0.5-0.8\right)}^{2}}=87.8\;\sim \;88$$

Categorical variables were tabulated and odds ratio for vaccine hesitancy was calculated. Fischer exact test was performed to analyze associations between background factors and the intention to be vaccinated. Pearson’s and Spearman’s correlation coefficients were performed were conducted to analyze correlations between fear of COVID-19, age, and number of symptoms after previous vaccination. The Kruskal-Wallis test was used to analyze the association between the ratings of COVID-19 disease fear and the intention to be vaccinated All tests were two-tailed and a *p*-value of less than 0.05 was considered significant.

## 3. Results

One-hundred and four medical students completed the survey. None of the students were vaccinated at the time of the survey. Students were of an average age of 29.2 years (23–39 years). Table 1 summarizes the students’ characteristics.

### 3.1. The Relation between Intention to Be Vaccinated against COVID-19 and Background Factors

Among the 104 students, 9 (8.7%) students stated that they had no intention of receiving the vaccine and 95 (91.3%) students intended to get vaccinated. Table 2 presents a comparison of several variables between these two groups of students. Analysis of potential relations between several background factors and the willingness to be vaccinated against COVID-19 showed no correlation between the intention to be vaccinated and gender, current pregnancy, health conditions, COVID-19 in family members, COVID-19 infection in the past year, pro-vaccine attitude, history of influenza, or history of vaccine adverse events in general. In contrast, a statistically significant association was found between previous experience with mandatory quarantine due to exposure to an individual with COVID-19 and objection to be vaccinated (*p* = 0.034); whereas 15.9% of the students who had been in mandatory quarantine in the past did not intend to be vaccinated, only 3.3% of the students who had not been in mandatory quarantine objected the vaccine. In addition, a statistically significant link between lack of knowledge about COVID-19 vaccine and objection to be vaccinated was identified (*p* < 0.001); over one-third (34.6%) of the students who indicated they lacked knowledge about the vaccine did not intend to be vaccinated, as compared with 0% of students who indicated their knowledge was partial or complete.

### 3.2. Links between Fear of COVID-19 and Background Factors

Table 3 presents correlations between age, number of symptoms from previous vaccines and fear of COVID-19 disease and its manifestations on health. Data were collected via the survey carried out by the students. There, they were asked to indicate whether they had experienced any side effects and which. Fear was rated by a scale from one to five (not at all—very high, respectively). A significant positive relation was found between the number of symptoms following previous vaccines and the fear of COVID-19 (rs = 0.272, *p* < 0.01). The more symptoms the student experienced from vaccines in the past, the higher their fear of COVID-19. No significant relation was found between student age and fear of COVID-19. No relationship was found between COVID-19 disease induced anxiety and the student’s academic year (*p* = 0.519).

### 3.3. Association between Fear of COVID-19 Disease and the Intention to Get Vaccinated

Table 4 presents the results of the Kruskal-Wallis test comparing the ratings of COVID-19 disease fear and the intention to be vaccinated. The rating was determined by an evaluation scale from one to five. According to Table 4, a statistically significant difference was evident in the fear of COVID-19 disease Between students who intent to be vaccinated and those who don’t (*p* < 0.001). The rating of the fear among students who did not intend to be vaccinated was higher compared to students who did intend to be vaccinated. Between students who intended to be vaccinated and those who did not (*p* < 0.001).

## 4. Discussion

Currently, Israel is among countries leading in rate of vaccinated persons for COVID-19 [1]. In December 2020, Israel initiated a national campaign to vaccinate its population. Israel’s Ministry of Health recommended initially a two-dose BNT162b2 vaccine series and since then, has approved a booster (3rd dose). The respective vaccine doses were rolled out approximately in parallel to Israel’s third and fourth waves of SARS-CoV-2 infection, waves with an average of thousands of new daily infections.

### 4.1. Background Factors That May Affect the Intention to Be Vaccinated against COVID-19

The World Health Organization labeled vaccine hesitancy among the top ten threats to global health [10]. Vaccine hesitancy has been a domain of concern globally for several decades. Vaccination hesitancy is prominent with current COVID-19 vaccines due to the inaccurate information circulating regarding the pandemic and vaccines [11]. This study set out to assess COVID-19 vaccine acceptability, hesitancy, and associated factors among medical students in Israel in the early stages of mass vaccination; the usual expectation from medical students is to have positive attitudes regarding vaccination.

The survey found that 91.3% of the participating Israeli medical students were willing to be vaccinated against COVID-19; only 9 (8.7%) were not. A similar acceptance level was reported among medical students in Italy (86.1%) and medical students in India (89.4%) [12,13]. In contrast, in a study performed in medical students in Uganda, acceptability was 37.3% and vaccine hesitancy was 30.7% (the data of the 33% left was not mentioned) [14]. The hesitancy regarding COVID-19 vaccination, according to our survey, in Israeli medical students was much lower than that reported among Egyptian medical students (46%) [15] and in Malta medical students (30.5%) [16]. This discrepancy can be explained by the variable impact of COVID-19 worldwide with various responses in countries affected mildly versus severely at the time of the survey. Another factor that is variable in the different countries and may affect the intention to get vaccinate is the proposed COVID-19 vaccine in each country, since each vaccine was developed in a different technology [17].According to a previous study among Egyptian medical students, if all types of vaccines were available in Egypt, the students would have preferred different vaccines, with 22% preferring the Pfizer-BioNTech vaccine, 7.1% the Oxford-AstraZeneca vaccine, 2.1% the Chinese Sinovac, 1.7% the Sputnik V, and 1.5% the Moderna vaccine, whereas 65.6% did not know what are the differences between the vaccines [18]. Other factors to consider are the magnitude of people’s trust in their government and the opinion towards vaccination of local leaders. According to previous studies, there is a link between people’s trust in their governments, and in their country’s experts and their will to get vaccinated [4,8,19]. Another study from India reported that one factor affecting vaccine hesitancy was mistrust in public health authorities [13].

Although most factors assessed here were not significantly different between the students who intended to be vaccinated and the student who planned to refrain, some differences were noted between the two groups. For example, males were slightly more likely to express their intention to be vaccinated than females (male, 92.5%; female, 90.6%). Similar finding was noted in a survey in the USA, with vaccine hesitancy odds higher for females (44% higher than males) [20]. The gender difference in vaccine hesitancy remains unclear but may be influenced by fear of COVID-19, perception of the severity of the disease, and fear of side-effects, infertility, and pregnancy complications. A previous survey reported that women tend to believe to conspiracy theories, more than men [21]. A study among Chinese students found that female students got less vaccinated although they had more fears to develop the disease, compared with male students [22]. Another possible reason for unwillingness to get vaccinated among women may be that women’s morbidity and mortality rates due to COVID-19 are lower compared to men [23]. Additionally, a previous study suggested that women’s reluctance may be derived from the rumors regarding fertility aspects following vaccination [4]. All pregnant women participating in the survey (*n* = 4) declared they intend to be vaccinated, while some of the non-pregnant woman indicated their intention not to receive vaccination. This unanimous response was likely due to fear of severe COVID-19 manifestations during pregnancy. The effect of COVID-19 infection on pregnancy remains minimally unknown. Nevertheless, pregnant women are generally considered a high-risk group for infectious diseases due to immunologic and physiologic changes [24]. While there is still no evidence that pregnant women are more susceptible to COVID-19 infection, pregnancy appears to worsen the clinical course of COVID-19 as compared to the course in non-pregnant individuals of the same sex and age [25,26]. Thus, it is possible that this data may have affected the pregnant students and motivated them to be vaccinated. However, our survey included only four pregnant women and more studies on the topic are still needed.

Most (8/9, 88.9%) of the students who did not intend to be vaccinated were healthy. The high vaccine acceptance among individuals with comorbidities is likely related to evidence that COVID-19 infection is more severe among people with co-morbidities as compared to healthy people [27]. These results align with studies demonstrating high intention of patients with common variable immunodeficiency to receive influenza vaccines and guidelines supporting COVID-19 vaccines in individuals with primary immunodeficiency [28,29].

Additionally, most (7/9, 77.8%) of the students who had no plans to be vaccinated had been quarantined in the past year. This finding was quite surprising, as people in quarantine lost work or study days, and experienced social isolation. A previous study among Indian medical students, most vaccinated students declared that one main reason for getting vaccinated was the will of getting back to their regular lives, including the frontal classes [13]. Therefore, we expected quarantined students to be more enthusiastic about potential vaccination. This topic is yet not fully understood but is likely influenced by the number of quarantines the individual experienced, or the reason for quarantine.

Another surprising finding was that most (66.7%) participants who had no plans to be vaccinated had a family member who was infected with COVID-19. We expected that people familiar with infected individuals would be more encouraged to receive the vaccine. However, their family members may have developed mild, moderate, or even asymptomatic disease, which may have lessened the perception of COVID-19 infection severity in the eyes of the students. A multicenter study reported that increased risk perception towards COVID-19 was associated with a higher likely uptake of the COVID-19 vaccine [30]. Of note, 7 out of the 9 students who had no plans to be vaccinated, did report on general support of vaccinations. Therefore, their intention not to get vaccinated against SARS-CoV-2 was apparently related to other factors.

Another factor that we investigated is the knowledge regarding the COVID-19 vaccine. In the beginning of December 2020 knowledge about long-term side effects and vaccine safety was still limited worldwide, while a few months later most of medical faculty students were vaccinated accompanied with knowledge about vaccine efficacy and safety. Although, as of December 2020, the date on which the medical students have replied the questionnaire, a non-negligible percentage (24.3%) replied they lacked knowledge regarding the vaccine. However, only 8.8% of the students felt worried about its side effects. Additionally, all those who had no plans to vaccinate felt they lacked knowledge regarding the vaccine. It is therefore imperative that medical students be encouraged to rely more on reliable sources. Therefore, targeted interventional educational campaigns are needed to combat misinformation and avoid low inoculation rates.

### 4.2. The Effect of Fear of COVID-19 on Vaccination Attitudes

The more symptoms students experienced from vaccines in the past, the higher their fear of COVID-19 disease (*p* < 0.01). Additionally, students with no plans to be vaccinated reported on higher fear scores as compared to those intending to receive the vaccine. This result was quite surprising since we expected that people afraid of a disease will be more cautious and more willing to vaccinate. In a study from Poland, medical students that wanted to get vaccinated, were influenced by fear of the disease [31]. According to a previous work, perception of increased risk for morbidity motivates vaccination [32]. On the other hand, a person who is in emotional stress due to a health threat may intend to take a preventive action. However, this intention may not necessarily be fulfilled [33]. Nevertheless, our finding was also seen in a previous study from Egypt, that presented an association between a high level of vaccine hesitancy and high perception level of getting infected. Additionally, only 35.1% of the vaccinated students declared they were afraid of getting infected [18]. One possible explanation that was reported in other studies is that participants who had been exposed to material supporting anti-vaccine conspiracy theories showed less intention to vaccinate despite fear of a disease, as compared to people who were not exposed to such materials [4,8]. A recent study mapping online views on vaccination concluded that “although smaller in overall size, anti-vaccination clusters manage to become highly entangled with undecided clusters in the main online network, whereas pro-vaccination clusters are more peripheral” [34].

A relatively positive attitude towards vaccination was also observed in our study. When compared to other vaccinations, such as HPV (32.1%) [35,36] and influenza (43.0%) [37], intention to vaccinate against COVID-19 higher, with most of the health care workers’ population fully vaccinated.

Furthermore, factors such as different beliefs are associated with the intention to be vaccinated against COVID-19, as has been shown in studies of vaccination acceptance for other diseases (e.g., flu [38] and HPV [35]). Similarly, as in another study, it was found that the intention to be vaccinated was associated with more positive general COVID-19 vaccination beliefs and attitudes, weaker beliefs that the vaccination could cause severe side effects or be unsafe, greater perceived information level, and greater perceived risk of COVID-19 [39].

Studies have reported that quarantine elicits negative psychological effects, including post-traumatic stress symptoms, confusion, and anger [40]. Stressors included longer quarantine duration, infection fears, frustration, boredom, inadequate supplies, inadequate information, financial loss, and even long-lasting effects [40]. These elements likely encourage individuals to be vaccinated (which is anticipated to decrease their chances of quarantine). Therefore, it was surprising to find that most of the students who did not intend to get vaccinated underwent a quarantine. Further studies should be performed in order to understand whether experiencing a quarantine affect the intention to get vaccinated.

In addition, due to medical students’ studies in a higher academic year, the surveyed student population showed a slight increase in the positive attitude regarding COVID-19 vaccine and less opposition to the vaccine. We can assume that since these students already had few months of experience in hospitals with COVID-19 disease and its manifestations, therefore about 90% present with intention to be vaccinated. In addition, their high intention might stem from their increased knowledge about the disease. A previous study from Poland found that students’ willingness to get vaccinated was affected from the year of studies; while students who wanted to get vaccinated immediately were at a median of three years, students who wanted to wait with vaccination had a median of two years [31]. The authors suggested that formation of attitudes and behaviors is more affected from active clinical practice than theoretical knowledge [31]. We also assume that students in healthcare-related schools might have a better ability to understand in depth the results of clinical trials on COVID-19 vaccines and, thus, have higher trust in and acceptance of such novel vaccines.

Furthermore, a statistically significant relationship was found between lack of knowledge about the COVID-19 vaccine and the intention to be vaccinated (*p* < 0.001); 36.9% of the students who indicated that they lacked knowledge about the vaccine did not intend to be vaccinated, compared to 0% of students who indicated that they were familiar with vaccine data, or lacked knowledge regarding the vaccine. These findings can be used to advocate the role of medical students in the dissemination of correct messages and attitudes regarding vaccination [7], in order to eradicate the phenomenon of vaccine opponents. Furthermore, we can assume that exposure to COVID-19 patients and even observing activities in COVID-19 hospital departments, may have led to different outcomes, and influenced student decisions regarding the vaccine.

Further, we assume that the majority of those expressing interest in being vaccinated were encouraged by the will for getting back to their routine lives, including studying in frontal classes (as previously found in a study from India) [13].

Participants may also have been influenced by exposure to COVID-19 vaccine-related topics in the media, friends, family, and university. In a previous study among 600 medical students in Uganda, the second most prevalent (53.5%) reason for not get vaccinated was negative information. The major source of this information was social media and friends. Other sources were television, newspapers, politicians, family, radio, and religious or cultural leaders [14]. A study from Israel among medical and nursing students found higher motivation for vaccination in students that were recommended to get vaccinate by their family, friends, colleagues, or supervisors [41].

Limitations of this study included the small sample size (104) and data collection at a single medical school, which may impact generalizability. In addition, the relatively small number of students with previous COVID-19 infection was likely a limiting factor on the results of the study. Another limitation is that the survey was conducted during the condition of BNT162b2 mRNA vaccine shot only. Further investigation should be performed nowadays, with other vaccines available.

## 5. Conclusions

Medical students represent the future generation, the doctors of tomorrow, and their attitude towards vaccines is highly important. With profound knowledge of vaccines and their benefits, they will be able to have a greater impact on the rest of the population. Overall, our medical students presented a high (91.3%) intention to receive the vaccine. The study highlights the need for education, awareness campaigns, and regulation of vaccine trials in order to further decrease COVID-19 vaccine hesitancy among medical students

## Figures and Tables

**Table 1:** Characteristics of study participants (*n* = 104).

Characteristic	*N* (%)
Gender	
Male	40 (38.5)
Female	64 (61.5)
Year in medical studies	
4	18(17.3)
5	41(39.5)
6	45 (43.2)
Health condition	
Healthy	93 (89.4)
With health disorders	11 (10.6)
Infection with COVID-19 during the last 12 months	
Yes	3 (2.9)
No	101 (97.1)
History of infection with flu in the last 5 years	
Yes	57 (55.3)
No	21 (20.4)
I don’t know	25 (24.3)

**Table 2:** Associations between background factors and the intention to be vaccinated.

Characteristic	Intention to Be Vaccinated		*p* Value
	Yes (*n* = 95)	No (*n* = 9)	
	N (%)		
Gender			1
Male	37 (92.5)	3 (7.5)	
Female	58 (90.6)	6 (9.4)	
Current pregnancy			1
Yes	4 (100)	0	
No	54 (90)	6 (10)	
Health condition			1
Healthy	85 (91.4)	8 (8.6)	
With health disorders	10 (90.9)	1 (9.1)	
History of quarantine for COVID-19			0.034 *
Yes	37 (84.1)	7 (15.9)	
No	58 (96.7)	2 (3.3)	
Previous COVID-19 infection in family member(s)			0.296
Yes	42 (87.5)	6 (12.5)	
No	53 (94.6)	3 (5.4)	
Infection with COVID-19 during the past 12 months			1
Yes	3 (100)	0	
No	92 (91.1)	9 (8.9)	
Pro vaccine attitude			0.242
Yes	86 (92.5)	7 (7.5)	
Partially	9 (81.8)	2 (18.2)	
History of influenza in the last 5 years			0.804
Yes	52 (91.2)	5 (8.8)	
No	20 (95.2)	1 (4.8)	
I don’t know	22 (88)	3 (12)	
Lack of information regarding the vaccine			<0.001 *
Yes	17 (65.4)	9 (34.6)	
Partially	31 (100)	0	
No	47 (100)	0	
Adverse events following previous vaccination			1
Yes	60 (90.9)	6 (9.1)	
No	35 (92.1)	3 (7.9)	

* *p* < 0.05.

**Table 3:** Correlations between fear of COVID-19, age, and number of symptoms after previous vaccination.

Characteristic	Average (SD)	Age	Number of Symptoms after Previous Vaccination	Fear of COVID-19
Age	29.2 (3.1)		0.084	0.109
Number of symptoms	2.6 (2.2)	0.053		0.272 **
Fear of COVID-19	2.5 (0.6)	0.179	0.298 **	

Underlined values represent results of the Spearman’s correlation coefficients were performed (rs). All other values were calculated using the Pearson’s correlation coefficient (rp); ** *p* < 0.01.

**Table 4:** Kruskal-Wallis test.

Vaccination Intention	N	Median (Range)	Average Rank	*p* Value *
No	9	3.2 (2.2–4.2)	83.3	<0.001
Yes	95	2.4 (1.2–3.6)	49.6	

* Determined using the Kruskal-Wallis test.

## Data Availability

The data presented in this study and the survey’s questions are available on request from the corresponding author.
